# Impact of age on the homing potential of ^89^Zr-radiolabelled CD8 + T cells

**DOI:** 10.1038/s41598-025-09237-y

**Published:** 2025-07-03

**Authors:** Jonas Bystrom, Melissa Pereira Da Costa, Amaia Carrascal-Miniño, Ahad Qureshi, George P. Keeling, Truc T. Pham, Kavitha Sunassee, Elizabeth C. Carroll, Conor Garrod-Ketchley, Johannes Schroth, Victoria S. K. Tsang, Rafael T. M. de Rosales, Samantha Y. A. Terry, Sian M. Henson

**Affiliations:** 1 Centre for Translational Medicine and Therapeutics, William Harvey Research Institute, Queen Mary University of London, London, EC1M 6BQ UK; 2 School of Biomedical Engineering & Imaging Sciences, King’s College London, St Thomas’ Hospital, London, SE1 7EH UK; 3 Department of Life Sciences, Atlantic Technological University Sligo, Ash Lane, Sligo, F91 YW50 Ireland

**Keywords:** Immunology, Imaging the immune system, Lymphocytes

## Abstract

The ability of CD8 + T cells to protect against infections and malignant transformations declines with age. Emerging technologies, such as total body positron emission tomography (PET) and radiotracers with long half-lives, offer new approaches to assess long-term cellular functional deficits in vivo. In this study, we radiolabelled human CD8 + T cells from both young and old individuals with zirconium-89 (⁸⁹Zr) and evaluated their distribution in vivo. ⁸⁹Zr-labelled CD8 + T cells were injected intravenously into NOD scid gamma mice, and their whole-body migration was tracked using PET imaging. Longitudinal PET imaging revealed that CD8 + T cells from older individuals accumulated in tissues at a slower rate compared to those from younger individuals and may have caused greater tissue damage. This impaired migration was associated with decreased cortactin expression and increased cholesterol levels in aged T cells, both of which have the potential to hinder cellular motility. This study established a method for labelling and tracking cryopreserved CD8 + T cells, though further research is needed to understand the differences in migratory behaviour between cells from young and older individuals.

**Supplementary Information:**

The online version contains supplementary material available at 10.1038/s41598-025-09237-y.

## Introduction

CD8 + T cells can be classified into distinct subsets based on their functional properties, homing preferences and differentiation status, typically defined by the expression of surface markers such as CD45RA, CD45RO, CCR7, CD28, and CD62L^1^. Naïve CD8 + T cells (N, CD45RA + CCR7 + CD28 + CD62L+) not yet encountering antigen, primarily circulate in lymphoid tissues. In contrast, central memory CD8 + T cells (CM, CD45RO + CCR7 + CD28 + CD62L+) while also residing in lymphoid tissues provide long-term immune protection by retaining the ability to proliferate rapidly upon antigen re-exposure^2^. Effector memory CD8 + T cells (EM, CD45RO + CCR7 − CD28 − CD62L−) are primed for rapid effector responses and circulate in peripheral tissues, albeit with reduced longevity compared to their central memory counterparts^2^. Finally, the terminally differentiated T cells that re-express CD45RA (EMRA, CD45RA + CCR7 − CD28 − CD62L−) comprise an important fraction of CD8 + T cells in the blood, exhibit features of replicative senescence and are significantly expanded in older individuals^3^. CD8 + EMRA cells tend to increase in blood and blood-rich sites such as the bone marrow, spleen and lungs as individuals age^4^. Thus, the accumulation of EMRA cells with age reflects a shift in the CD8 + T cell compartment contributing to the functional alterations observed in adaptive immunity during ageing^5^.

Given these systemic changes that occur with age, it is increasingly important to employ advanced imaging techniques that can capture the dynamic interplay of immune cells throughout the body. Total body positron emission tomography (TB-PET) imaging allows for clinical non-invasive total body imaging with a 10- to 40-fold increase in sensitivity compared to conventional PET systems^6,7^. TB-PET therefore provides the means to analyse immune cell localisation in the whole body in detail, using small numbers of radionuclide-labelled cells^8,9^. Using radionuclides for PET imaging makes the detection and quantification of discrete cell populations, including radiosensitive T cells, within defined tissue locations possible^10^.

PET radionuclides with a long half-life, such as ^89^Zr (*t*_1/2_ = 78.4 h), can be used for (pre)clinical imaging and enable the detection of cells longitudinally from repeat PET scans. Indirect radiolabelling through ^89^Zr-labelled antibodies has been used to demonstrate the role of CD8 + T cells in tumour immunity and their response to therapy^9,11^. However, direct labelling of immune cells with ^89^Zr provides increased sensitivity and specificity^12,13^ and can be achieved through the chelation of ^89^Zr with four oxine (8-hydroxyquinoline) molecules to generate a metastable complex that can cross cell membranes. This complex dissociates inside cells, depositing ^89^Zr bound to intracellular proteins^12,13^. A usual caveat with direct labelling is that lymphocytes are prone to radiation damage, senescence, and cell death^14,15^. The advent of TB-PET has meant that T cells can now be labelled with ^89^Zr at lower activity levels without compromising detectability; this approach showed no reduction in cytokine production or migration when subjected to 70 kBq/10^6^ cells^16^. However, it remains unexplored whether CD8 + EMRA cells responds differently to cell labelling and whether homing abilities are altered. This is important as most chronic diseases requiring analysis by PET generally occur at an older age^11^. Therefore, it is of interest to determine whether the build-up of CD8 + EMRA T cells with age influences the ability of ^89^Zr labelling for imaging.

We show here the radiolabelling efficiencies and retention of human CD8 + T cells isolated from young and older individuals with [^89^Zr]Zr(oxinate)_4_ and assess the distribution of these radiolabelled CD8 + T cells in vivo. We found that cryopreservation does not influence the ability of CD8 + T cells to be labelled with ^89^Zr and that cryopreserved ^89^Zr-CD8 + T cells were readily detected using PET imaging for at least up to three days in mice. Longitudinal assessment by in vivo PET scanning indicated retention of ^89^Zr within CD8 + T cells albeit it with different migratory patterns observed when using young and old T cells. This study suggests that the age of CD8 + T cells should be considered in future longitudinal PET imaging studies using in vivo cell labelling as it may influence the migratory capacity of the studied cells.

## Materials and methods

### Animals

Female Nod *scid* gamma (NSG, NOD.Cg-*Prkdc* ^scid^ *IL2rg*^*tm1Wjl*^/SzJ) mice aged 6 weeks were purchased from Charles River (UK), with an average body weight of 20.7 ± 1.5 g; mean ± SD as provided by the supplier. Only female NSG mice were used in this study as they were found to better support engraftment of human immune cells^17^.

### Ethical approval

Our clinical protocol was approved by the West London & GTAC Research Ethics Committee (20/PR/0921) and all methods were carried out in accordance with approved guidelines and regulations. Written informed consent was obtained from all subjects. We recruited healthy volunteers in two age ranges, young: 23–45 years of age (*n* = 20), and old: 69–82 years of age (*n* = 16). Animal experiments were approved by the UK Home Office under The Animals (Scientific Procedures) Act (1986), with local approval from King’s College London Animal Welfare and Ethics Review Body. All procedures complied with relevant guidelines and regulations and have been reported in accordance with the ARRIVE guidelines.

### Cell isolation

Blood (30–60 mL) was obtained from healthy young or older individuals. Peripheral blood mononucleated cells (PBMCs) were isolated using Ficoll Paque (GE Healthcare). PBMCs were resuspended in foetal bovine serum (FBS) with 10% dimethylsulfoxide (DMSO) and stored in liquid nitrogen or used directly. Cryopreserved PBMCs were thawed, washed once using RPMI (Merck Millipore), resuspended in RPMI culture medium, counted and stored at 37 °C 1 h before the labelling procedure. CD8 + T cells were isolated from PBMCs using magnetic beads (Miltenyi) and magnetic column as has been described before^18^. CD8 + T cells from cryopreserved samples were isolated upon thawing and then treated as the cryopreserved PBMCs prior to labelling.

### Cell culture

1 × 10^6^ radiolabelled or vehicle-treated PBMCs were cultured for up to 4 days using RPMI supplemented with 10% FBS, L-glutamine (2 mM), penicillin and streptomycin (Sigma) and 1 ng/mL IL-2 (BioLegend).

### [^89^Zr]Zr-oxine synthesis and cell labelling

^89^Zr was chelated with oxine forming [^89^Zr]Zr(oxinate)_4_ (Perkin Elmer) as previously described. PBMCs or separated CD8 + T cells were washed with Phosphate Buffered Saline (PBS) (Ca^2+^/Mg^2+^ free) (Merck Millipore), resuspended in PBS and mixed with [^89^Zr]Zr(oxinate)_4_ with a volume ratio not lower than 30:1 (cells: [^89^Zr]Zr(oxinate)_4_), using 3-20 × 10^6^ cells and 5-100 mBq/cell in various experiments. After radiolabelling, cells were washed using 50 mL PBS and quantified using a haemocytometer. Radioactivity in the pellets and supernatants of centrifuged and washed cells was then counted using a Wallac Wizard gamma counter to determine cell-associated radioactivity. The percentage of injected activity (%IA) present in cells was then calculated.

### In vivo PET imaging of CD8 + T cells

^89^Zr-labelled CD8 + T cells were injected intravenously into NOD scid gamma (NSG) mice (3 × 10^6^ cells/animal in 100 µL PBS, 63 ± 44 kBq, single CD8 + T donor per experiment) under anaesthesia (1.5–2.5% isoflurane in oxygen). Three hours post-injection, mice were re-anaesthetised and placed in a preclinical nanoPET/CT scanner (Mediso) where anaesthesia was maintained, and the bed was heated to maintain a normal body temperature. Two hours of PET acquisition (1:5 coincidence mode; 5 nanoseconds coincidence time window) were followed by CT. PET-CT was repeated at t = 24 and 72 h (2 and 3 h, respectively). Kinetic information from the PET/CT images were reconstructed using a Monte Carlo-based full-3D iterative algorithm (Tera-Tomo, 400–600 keV energy window, 1–3 coincidence mode, 4 iterations and subsets) at a voxel size of (0.4 × 0.4 × 0.4) mm^3^ at 2 frames per minute and corrected for attenuation, scatter, and decay. Images were co-registered and analysed using VivoQuant v.3.0 (InVicro LLC) capturing maximum intensity projection (MIP) and transverse plane images. Image analysis was conducted on the summed images for each time point with background, not associated with anatomy, being removed manually. Spherical volumes of interest (VOIs) were placed on organs based on the CT image when tissue, e.g. spleen, was not visible. Percentage injected activity (%IA) was calculated as follows %IA = (Activity remaining at scan time / Initial injected activity) * 100 to assess overall injected activity in each animal and %IA/mL to determine injected activity distribution and concentration in selected organs.

### Ex vivo biodistribution

Mice from imaging studies were used for biodistribution studies at 72 h post-injection. After culling, organs were dissected, weighed, and γ-counted together with standards prepared from a sample of injected material. The percentage of injected activity per gram (%IA/g) of tissue was calculated.

### Flow cytometry

Flow cytometric analysis was carried out using the following antibodies: anti-ADAM28 (AB28292; Abcam), anti-cortactin (AB2547273; ThermoFisher), anti-rabbit IgG Cy3 (Poly4064), anti-CD8 PerCP (SK1), anti-CD45RA APC (HI1000) and anti-CCR7 PE-Cy7 (G043H7) from BioLegend. Membrane cholesterol content was measured by incubating PBMCs with 50 µg/mL filipin III (Sigma) for 2 h at room temperature, followed by staining with phenotypic T cell markers listed above.

All samples were analysed using a FACS Melody (BD Biosciences) and the resulting data was examined using FlowJo software (BD Biosciences).

### In vitro T cell migration

Human umbilical vein endothelial cells (HUVEC; PromoCell) were added to 48-well plates coated in 1% porcine gelatine (Sigma) at a concentration of 2.5 × 10^4^ cells/well in M199 media (ThermoFisher). Once a monolayer had formed the HUVECs were stimulated with 10 ng/mL TNF-α and IFN-γ (BioLegend) for 24 h. CD8 + T cells were isolated by negative selection, followed by isolation of effector memory (EM) and effector memory CD45RA re-expression (EMRA) T cells using CD27 and CD45RO beads (Miltenyi). Isolated T cell populations were added to the stimulated HUVECs at a concentration of 5 × 10^5^ cells/well in M199 media. T cells were left for 8 min after which time non-adherent cells were removed. Transmigration was assayed for 30 min at x10 objective with an Olympus iX81 with a Hamamatsu Orca ER digital camera, with frames taken every 30 s. Distance and speed were calculated using Image J (Fiji).

### Immunofluorescence staining

Tissues were snap-frozen in OCT media and the radioactivity allowed to decay to background. Sections were cut by the Bart’s Cancer Institute Pathology Services Lab at QMUL and permeabilised in 1% Triton X-100 in PBS with 0.1% Tween 20 (PBST) for 10 min at room temperature. The sections were blocked with 1% BSA in PBST and incubated with the indicated primary or conjugated antibodies for 1 h at room temperature, followed by the secondary antibodies for 1 h. Conjugated antibody used: anti-CD8-FITC (1:100; BioLegend; SK1). Primary antibody used: anti-laminin (1:400; Sigma; LAM-89). Secondary antibody used: anti-rabbit IgG Cy3 (1:2000; BioLegend; Poly4064).

In situ direct DNA Fragmentation (TUNEL) staining was performed according to the manufacturer’s instructions (Abcam). Following permeabilization with 1% Triton X-100 and 0.1% Tween 20 in PBS for 10 min and blocking with 1% goat serum and 0.1% Tween 20 in PBS for 30 min at room temperature, the sections were washed with the Abcam TUNEL-kit wash buffer. The sections were then incubated with the staining solution (TdT Enzyme and FITC-dUTP diluted in reaction buffer and ddH_2_O) for 60 min at 37 °C and with Propidium Iodide (PI)/RNase solution for 30 min at room temperature. Sections were mounted in Vectashield Plus Antifade Reagent (Vector Laboratories) and visualized and analysed using the Cytation 5 (Agilent). After a cell mask was applied, apoptotic cells were identified as a green staining over an orange-red PI counter-staining (Ex/Em = 469/525 nm for FITC, and 488/623 nm for PI).

Normalised apoptotic cells were calculated as follows: Normalised apoptotic cells = TUNEL & PI/RNAse positive cells / Total area x1000.

### Statistical analysis

Statistical analysis was performed using GraphPad Prism version 9.5. A Mann-Whitney U test was used to compare differences between groups and Wilcoxon signed rank test within groups, as data was not normally distributed. ANOVA followed by Tukey post hoc testing was used when comparing more than two groups. Data was expressed as mean ± SD and statistically significant differences were represented using the following notation: * = *p* < 0.05 and *** = *p* < 0.005.

## Results

**Fig. 1:**
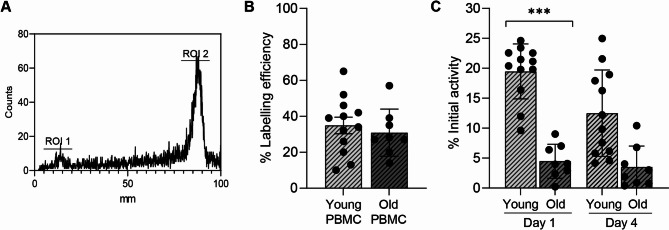
(**A**) Example of radio-TLC to determine [^89^Zr]Zr-(oxinate)_4_ chelation efficiency, with region of interest (ROI) region 1 being free ^89^Zr (5.9%) and ROI 2 being [^89^Zr]Zr-(oxinate)_4_, (94.1%). (**B**) [^89^Zr]Zr-(oxinate)_4_ cell labelling efficiency with young or old cryopreserved PBMCs. (**C**) Percentage ^89^Zr remaining associated with cells based on initial cell-bound (from B) activity following cell culture at day one and four in young or old cryopreserved PBMCs.

A previously established kit formulation was used to create [^89^Zr]Zr(oxinate)_4_ with a radiochemical yield of 82.3 ± 7.7% (Fig. 1A)^13^. Cryopreserved PBMCs from young and older participants were successfully labelled with [^89^Zr]Zr(oxinate) and found not to differ in their labelling efficiency post-labelling (Fig. 1B). The labelling efficiency was highly variable, but in line with what others have reported for primary immune cells^19,20^. Therefore, we concluded that neither cryopreservation nor age influenced cell labelling efficiency. However, differences between young and old cryopreserved PBMCs did become apparent when the cells were cultured. Cryopreserved PBMCs from young participants retained significantly more of the initial activity than cells isolated from older participants, assessed at days one and four post cell labelling (Fig. 1C). This may reflect the fact that membrane repair becomes impaired with age^21^. Overall, the data shows no age-related effect on the ability of ^89^Zr to radiolabel immune cells, although PBMCs from older volunteers retained less radionuclide during in vitro culture.

**Fig. 2:**
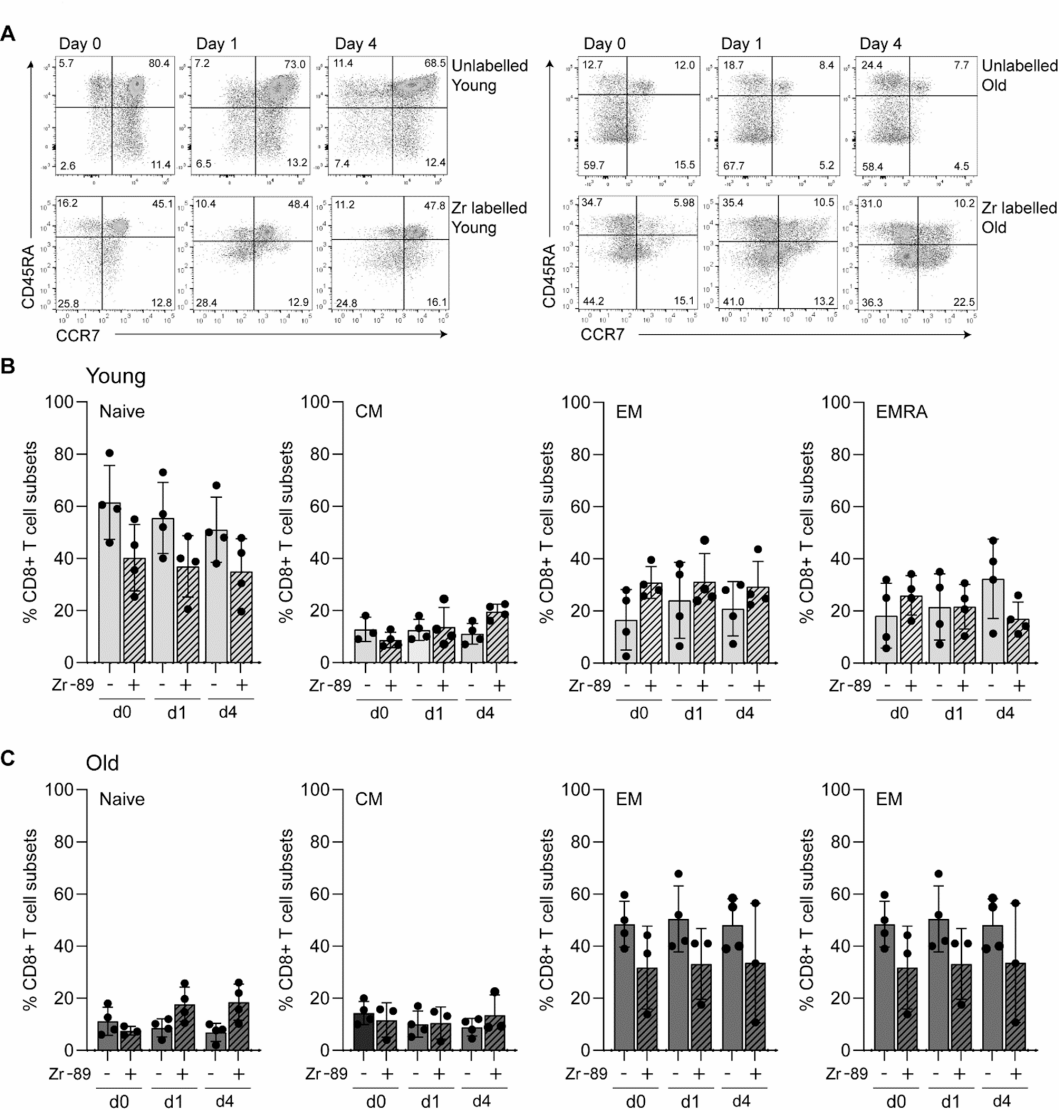
(**A**) Representative flow cytometry plots showing CD45RA/CCR7-defined CD8 + T cell subsets with and without ^89^Zr radiolabelling taken from young and older people at baseline and then following one and four days of culture. Naïve: CD45RA + CCR7+, central memory (CM): CD45RA-CCR7+, effector memory (EM): CD45RA-CCR7-, EMRA: CD45RA + CCR7-. (**B**) The proportions of each of the CD8 + T cell subsets taken from young individuals and (**C**) older individuals.

Given that CD8 + T cells are phenotypically diverse, we next examined their heterogeneity by classifying them into four distinct populations based on the expression of CD45RA and CCR7: CD45RA⁺CCR7⁺ naïve T cells, CD45RA⁻CCR7⁺ central memory (CM) T cells, CD45RA⁻CCR7⁻ effector memory (EM) T cells, and CD45RA⁺CCR7⁻ EMRA T cells (Supplementary Fig. 1). We assessed whether CD8 + T cell subset composition differed following ^89^Zr-labelling and we found radiolabelling caused no changes to the relative proportions of CCR7/CD45RA-defined CD8 + T cells when compared to unlabelled cells (Fig. 2). Although CD8 + EMRA T cells were more numerous in older individuals the relative subset distribution was not affected by ^89^Zr-labelling (Fig. 2B, C). These results suggest that ^89^Zr-labelling did not influence CD8 + T cell differentiation.

**Fig. 3:**
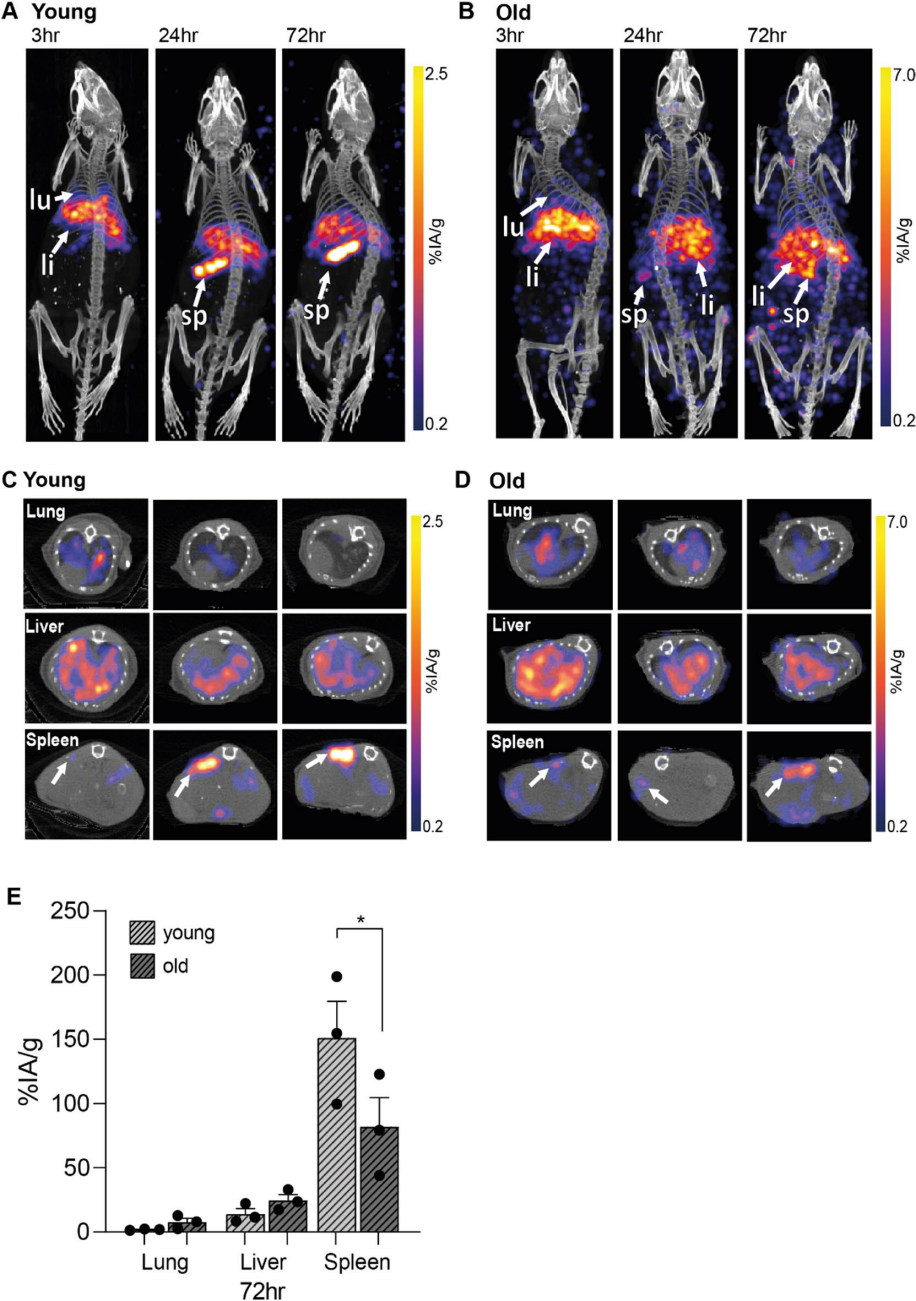
Representative images of NSG mice injected intravenously with ^89^Zr-labelled 3 × 10^6^ CD8 + T cells from a young, (**A**) an older individual, (**B**). Mice were subjected to whole-body preclinical PET at 3 h (2 h scan), 24 h (2 h scan) and 72 h (3 h scan). Transverse sections at time-points 3, 24 and 72 h highlighting regions with lungs, livers and spleens using ^89^Zr labelled cells from young, (**C**) and old individuals, (**D**). White arrows indicate lung (lu), liver (li) and spleen (sp). Data was reconstructed and analysed using Vivoquant. (**E**) Ex vivo biodistribution of ^89^Zr-labelled CD8 + T cells isolated from young and old individuals 72 h after CD8 + T cell administration.

To determine whether age would influence PET imaging studies, 3 × 10^6^ CD8 + T cells from young and older individuals were labelled with [^89^Zr]Zr-(oxinate)_4_ and injected into NOD scid gamma (NSG) mice with an average activity of 63 ± 44 kBq (Fig. 3). However, owing to the loss of ^89^Zr from aged CD8 + T cells (Fig. 1C), significantly less activity was inject when using these cells (Young CD8 + T cells: 105 ± 32 kBq; Old CD8 + T cells: 22 ± 17 kBq). Despite this difference, PET imaging successfully detected CD8 + T cells from both age groups, with CD8 + T cells initially localising to the lung and liver (3 h, Fig. 3A-D). While young and old CD8 + T cells gradually accumulated in the spleen, a higher proportion of old CD8 + T cells remained in the liver and lung (Fig. 3C, D). The organ weight and injected radioactive activity were normalised for all imaging timepoints. Biodistribution showed the highest concentration of ^89^Zr labelled CD8 + T cells were found in the spleen (Fig. 3E). However, CD8 + T cells from older individuals accumulated in the spleen at a significantly lower rate compared to cells isolated from young individuals (Fig. 3E). Uptake in other organs showed no major differences between the two groups, suggesting that the radiolabel remains stably associated with T cells in vivo (Supplementary Fig. 1B). Additionally, the absence of significant bone uptake, where free ^89^Zr typically accumulates^22^further indicates cell-bound activity rather than free ^89^Zr. It is possible that free ^89^Zr may be distributed diffusely throughout the body, resulting in low-level, sub-threshold activity that is not readily detectable in individual organs. However, we believe that the loss of ^89^Zr occurred prior to injection, suggesting an age-related decline in the migratory capacity of CD8 + T cells from older individuals.

**Fig. 4:**
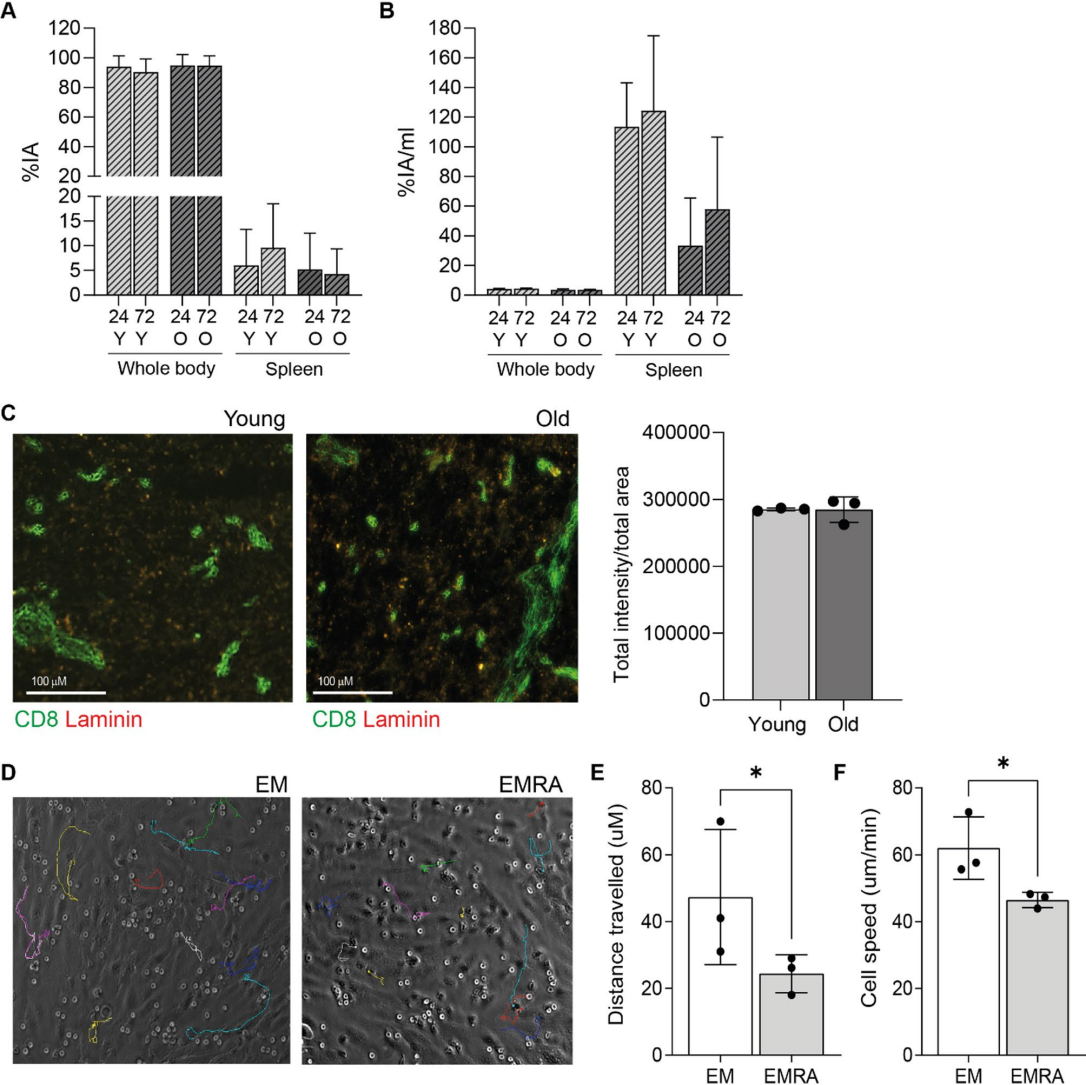
(**A**) Graphs showing the image-based analysis of percentage injected activity (%IA) and (**B**) injected activity/mL, calculated for the whole-body and spleen tissue VOIs of 2 mice per group at 24 and 72 h after the administration of radioactive CD8 + T cells from young and old individuals. (**C**) Immunofluorescent staining visualising CD8 + T cells (green) and laminin (red) in spleen sections from animals receiving ^89^Zr radiolabelled CD8 + T cells from young or old individuals. Graph shows the quantification of the total CD8 intensity over the total area of the section. (**D**) Brightfield images showing the migratory paths of CD8 + EM and EMRA T cells when co-cultured on stimulated HUVEC. (**E**) Averaged data from three experiments showing the distance travel and (**F**) the speed of travel of CD8 + T cell subsets. Both calculated using ImageJ.

This altered migratory potential of aged T cells was further seen when the percentage injected activity (%IA) was calculated from the serially performed PET scans (Fig. 4). Most of the radioactivity was still circulating 24 h post-administration, with approximately 99% of the injected activity being found in the whole-body volume of interest (VOI). While this is consistent with the normal biodistribution and pharmacokinetics of ^89^Zr-oxine labelled cells^23^it was noticeable that a higher %IA was present in the spleen tissue receiving CD8 + T cells from young individuals. At 72 h post-administration the %IA in the whole body remained high, however, the highest %IA again came from spleens receiving CD8 + T cells from young people (Fig. 4A). The distribution and concentration of the radioactivity in the organs were measured to determine the percentage of injected activity per mL, allowing for a more accurate comparison when normalising the IA by the tissue volume and to consider the lower labelling efficiency of CD8 + T cells from older individuals. When this was performed, we again found that %IA per mL was higher in the spleens that received CD8 + T cells from young individuals (Fig. 4B). Additionally, kinetic data showed that ^89^Zr-labelled CD8 + T cells from older individuals initially accumulated in the liver at higher levels compared to those from younger individuals. However, this difference diminished over time (Supplementary Fig. 1C). In contrast, both young and old ^89^Zr-labelled CD8 + T cells gradually accumulated in the spleen, with a notably higher proportion of CD8 + T cells from younger individuals observed at later time points (Supplementary Fig. 1C). This suggests that CD8 + T cells from young individuals migrated more rapidly compared to those from older individuals. However, when we evaluated immunofluorescence staining of CD8 + T cells in the spleens of animals receiving ^89^Zr-oxine labelled young or old T cells, we found no differences in the amount of CD8 + T cells present (Fig. 4C). Since all mice received equal numbers of CD8 + T cells, the aged CD8 + T cells in this closed system will eventually accumulate in proportions similar to those of CD8 + T cells from young individuals, given sufficient time.

To confirm whether the observed difference in migratory capacity was not exacerbated by the disparity in radiolabelling efficiency and PET signal, in vitro transmigration assays were performed. We isolated CD8 + EM and EMRA T cell subsets, to represent the build-up of highly differentiated T cells with age. These T cell subsets were then co-cultured with stimulated HUVECs, and their movement was tracked for over 30 min (Fig. 4D, Supplementary Movie 1, 2). EM cells were found to migrate further (Fig. 4E) and faster (Fig. 4F) than EMRA T cells. This suggests that the differences observed in the PET imaging of CD8 + T cells from young or old individuals were not solely attributable to labelling effects but were indeed influenced by their migratory capacity.

**Fig. 5:**
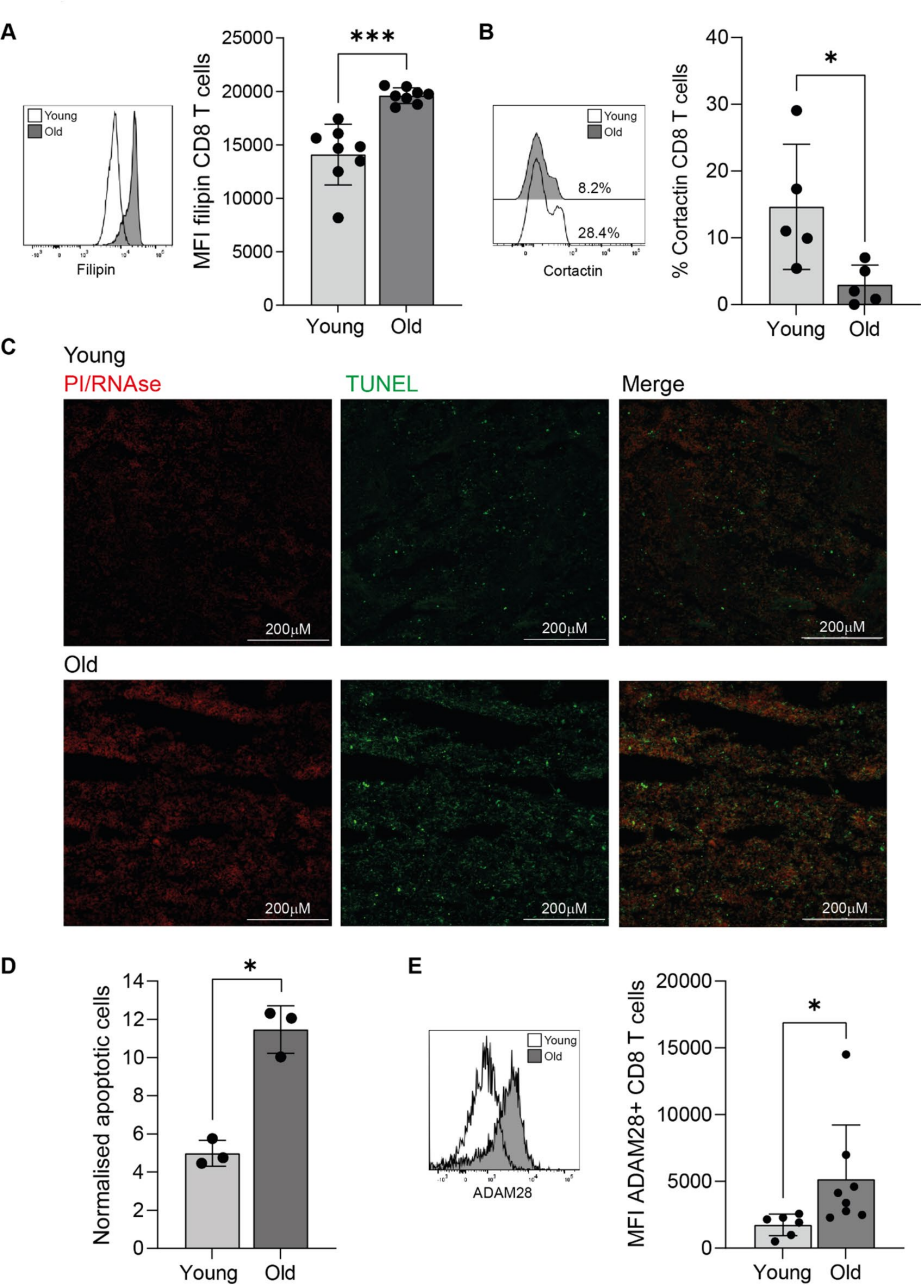
Representative histograms and graph of (**A**) filipin and (**B**) cortactin expression in CD8 + T cells from young and old individuals. (**C**) TUNEL staining (green), PI/RNAse (red) and merged image in spleen sections from animals receiving ^89^Zr radiolabelled CD8 + T cells from young or old individuals. (**D**) Graph shows the quantification of the number of apoptotic (TUNEL positive) cells normalised to the total area of the section. (**E**) Representative histogram and graph of ADAM28 expression in CD8 + T cells from young and old individuals.

The mechanisms underlying the observed differences in migration may result from a combination of factors, including an increase in membrane cholesterol content. Cholesterol plays an important role in modulating membrane fluidity. Increased cholesterol levels can lead to stiffer, less flexible membranes that can impair the ability of CD8 + T cells to deform and migrate efficiently. We show here an increased filipin III expression in CD8 + T cells from old individuals (Fig. 5A). We also find the expression of cortactin to be reduced on CD8 + T cells from old individuals (Fig. 5B). Cortactin is involved in cell motility by regulating the assembly of branched actin filaments, essential for pushing cells forward. Lower cortactin expression may reduce the ability of CD8 + T cells to generate the necessary forces for movement through tissues, resulting in slower migration.

Finally, we assessed the impact of the reduced migratory speed of CD8 + T cells from older individuals, as we and others have previously shown that these cells exhibit a senescence-associated secretory phenotype (SASP)^24,25^. This pro-inflammatory secretome is characterised by increased secretion of cytokines, chemokines and proteases that can contribute to chronic inflammation and tissue dysfunction. Given these properties, we investigated whether aged CD8 + T cells could exacerbate tissue damage in the spleen using TUNEL staining. Apoptotic cells, identified as double-positive for TUNEL staining and PI/RNase staining, appeared to be more prevalent in the spleens of animals that received old CD8 + T cells compared to those that received young CD8 + T cells (Fig. 5C, D). We can infer that this increase in apoptosis is not due to the ^89^Zr radiolabelling, as mice that received CD8 + T cells from old individuals exhibited lower radioactivity. Instead, the potential increase in TUNEL staining likely reflects inherent differences between young and old CD8 + T cells. As T cells age, they develop a highly inflammatory secretome, which includes proteases such as ADAM28 (Fig. 5E). The elevated expression of ADAM28 may contribute to the higher burden of apoptotic cells in spleens of mice receiving CD8 + T cells from old people.

Overall, these findings suggest that cryopreserved CD8 + T cells can be effectively radiolabelled with zirconium. Although aged CD8 + T cells lose the radiolabel more quickly than those from young individuals, they remain detectable via PET scanning. Furthermore, aged CD8 + T cells exhibit slower migration into the tissue, where they contributed to greater stromal damage.

## Discussion

PET provides whole-body imaging, essential for monitoring the distribution, migration, and accumulation of immune cells in various tissues. This can be particularly useful during ageing where age-associated changes to T cell compartments exhibit site-specific and subset-specific dynamics. In this study we used PET imaging to gained important insights that will enhance the tracking of immune cell subpopulations. Our findings suggest that cryopreserved CD8 + T cells can be effectively radiolabelled with zirconium-89. The high specificity of directly labelled cells offers an advantage over radiolabelled antibodies, where nonspecific binding may occur leading to image quality degradation^13,26^. In our study, we found the labelling efficiency of primary immune cells to be highly variable, but in line with previous reports^19,20^. We believe this variability was not due to differences in immune cell subtypes between individuals^12^but was more likely a result of the radiolabelling conditions, including isotope type, buffer composition, pH, temperature, cell type and their degree of cellular stress^27^. It should also be noted that ^89^Zr labelling efficiency of primary immune cells is lower than that demonstrated for CAR-T cells, due to their enhanced activation increasing cellular metabolism and membrane dynamics, improving membrane diffusion and intracellular protein binding^16^.

Despite aged CD8 + T cells losing the ^89^Zr radiolabel more rapidly than those from young individuals, they remain detectable via PET scanning. This age-associated decline in ^89^Zr retention may result from disruptions in ^89^Zr-intracellular protein complexes leading to increased release of free ^89^Zr. Either through impaired protein synthesis, altering protein binding affinity, or membrane dysfunction causing leakage of intracellular components, including unbound ^89^Zr^21,28^. These factors may explain the observed differences in PET signal, with aged cells displaying higher whole-body retention of free ^89^Zr and reduced image quality due to an increased signal-to-noise ratio. The advent of total-body PET with the ability to detect cells with a high sensitivity while using less injected radioactivity^29^ may present a valuable advantage for directly labelled cells, particularly in older individuals, by enhancing detection despite reduced radiolabel retention.

Tissue distribution of ^89^Zr-radiolabelled CD8 + T cells suggested that there was an age-dependent difference in organ homing with an alteration in migration dynamics between young and old CD8 + T cells. Aged CD8 + T cells remained to a larger extent in the liver, while young cells more efficiently infiltrated the spleen of the NSG mice. We also observed that CD8 + T cells taken from younger individuals migrated at a faster rate than CD8 + T cells from older people, a finding that is corroborated in other tissues^30^. Fibroblasts treated to induce senescence either through the upregulation of p16 or with mitomycin showed a slower rate of migration^30^. These changes were primarily due to the reduced capacity to synthesize a functional extracellular matrix (ECM) network, particularly altered expression of ECM-related genes including collagens, lysyl oxidases, and matrix metalloproteinases. We have shown previously that CD8 + EMRA T cells express high levels of collagens and proteases^24^and here that CD8 + T cells from older individuals express higher levels of ADAM28, a membrane-bound proteinase that can be released through proteolytic cleavage. The soluble form of ADAM28 has been reported to enhance the α4β1-dependent cell adhesion and therefore can influence T cell adhesion and migration^31^. When taken together with increased cholesterol, which can influence cell migration by impacting cytoskeletal organization and adhesion to the ECM^32^ and reduced cortactin, which regulates actin dynamics and cell motility all point towards a reduced motility.

The highly inflammatory secretory nature of senescent cells, termed the senescence-associated secretory phenotype (SASP), disrupts the stromal microenvironment and modulates the migration of surrounding non-senescent cells^33^. By altering the ECM, increasing adhesion molecule expression and influencing chemotactic signalling, the SASP creates conditions that can either impede or redirect immune cell migration contributing to age-related changes in immune cell trafficking^34^. The elevated secretion of matrix metalloproteinases, such as ADAM28 shown here, along with inflammatory cytokines, accelerates ECM degradation compromising tissue integrity, weakening the stromal barrier and increasing permeability. This breakdown of structural components not only facilitates aberrant immune cell infiltration but also disrupts normal tissue homeostasis. Additionally, SASP-driven ECM remodelling can promote fibrosis and excessive collagen deposition, leading to tissue stiffening that further restricts T cell migration^35^. These combined effects create a dysfunctional microenvironment that hinders effective immune surveillance and may contribute to chronic inflammation and impaired tissue repair.

In summary, work presented here highlights the importance of considering cellular senescence when applying specific diagnostic modalities. In particular, these findings have implications for human CD8 + T cell tracking and detailed localisation preference using TB-PET imaging.

## Electronic supplementary material

Below is the link to the electronic supplementary material.

Supplementary Material 1

Supplementary Material 2

Supplementary Material 3

## Data Availability

Data will be made available upon reasonable request to Prof Sian Henson.
