# Supplementary material for: Impact of age on the homing potential of 89Zr-radiolabelled CD8 + T cells
Source: Sci Rep. 2025 Jul 3;15:23801. doi: 10.1038/s41598-025-09237-y (PMC12229634; doi:10.1038/s41598-025-09237-y)

**Movie 1.** 2.5 x 10^4^ EM cells/well incubated with TNF-α and IFN-γ stimulated HUVEC, imaged every 30 seconds over 30 minute period. ImageJ was used to track the cells.

**Movie 2.** 2.5 x 10^4^ EMRA cells/well incubated with TNF-α and IFN-γ stimulated HUVEC, imaged every 30 seconds over 30 minute period. ImageJ was used to track the cells.

**Supplementary Figure 1. A.** Dot plots showing the gating strategy used to classify CD8+ T cells subsets defined using CCR7 and CD45RA. **B.** *Ex vivo* biodistribution of ^89^Zr-labelled CD8+ T cells isolated from young and old individuals 72 hours after CD8+ T cell administration. **C.** Activities (%ID/g/mm^3^) in ROIs representing Spleen, Liver and Lung at time points, 3hr, 24hr and 72hr.


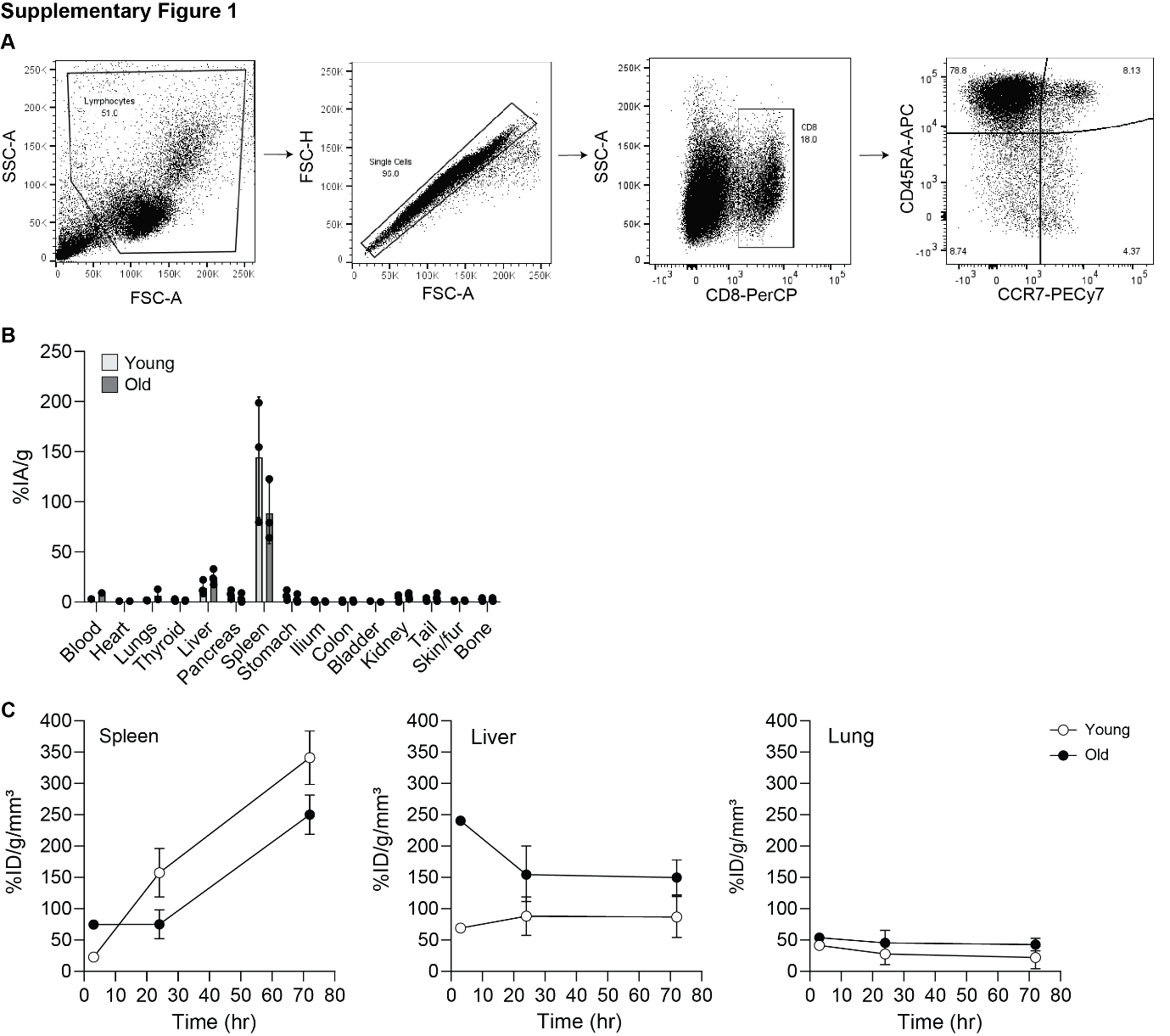

Supplement: Supplementary file 3 — Supplementary Material 3 [file 41598_2025_9237_MOESM3_ESM.docx]
